# Progress in Pediatrics

**Published:** 1901-12-28

**Authors:** 


### Progress in Pediatrics.

(*Continued from page* 203.)

**Boiled versus Unboiled Milk.**—A discussion on this subject has taken place in the columns of the *Lancet*. The subject was first raised by Dr. Clement Dukes,[6] of Rugby, who holds that the nutritive value of milk is very largely diminished by boiling or sterilising. Unfortunately, he does not bring forward any direct evidence in support of his views, save that in a large public school, with which he has been connected for 30 years, the milk has always been consumed in a raw state, and no bad effects have followed. This, of course, supports his view that raw milk is not necessarily dangerous, but it does not prove that boiled milk is of inferior nutritive value. He holds that more attention should be paid to the purity of dairies and the means of transit of the milk, and less to the boiling. Unfortunately, also, in his remarks and in those of the writers who follow him, the exact meaning of boiled milk is not stated, so that the different processes of bringing to the boil, boiling for one minute, or for 30 minutes, or placing the milk in boiling water, seem all to be lumped together. It is self-evident that in referring to boiled milk a clear statement should always be made as to the process and the length of time occupied. Dr. G. Variot[7] denies that the sterilising of milk, even at 115° C., makes t lose its nutritive qualities, and finds, on the contrary, that on its exclusive use they can, in Paris, bring up healthy children and improve atrophic ones. Scurvy and rickets are extremely rare, and the only undesirable results not unfrequently noted are anæmia and habitual constipation. Dr. Thomas Dutton[8] thinks that Dr. Dukes' practice may be suited to a country place like Rugby, but is entirely unsuited to any large city where the risks of contamination are so great. Dr. Fred. J. Smith[9] follows on the lines of Dr. Dukes, and makes the sweeping statement that in his out-patient department at the London hospital "it is simply heartrending to see the number of helpless creatures who are made peevish, fretful, and even seriously ill by the pernicious effects of boiled milk on their nutrition." One cannot help wondering when this epidemic of milk-boiling broke out amongst the mothers in the East End of London, for it is not common amongst mothers attending children's hospitals elsewhere. As regards the "pernicious effects of boiled milk" Dr. Robert Hutchison,[10] of the Great Ormond Street Hospital, writes that he always orders the milk to be boiled and rarely finds any difficulty in obtaining the normal increase of weight. This is no mere general impression, for his patients are weighed regularly once a week and an exact diet prescribed. He further points out that a distinction must be drawn between

*digestibility* and *absorbability*, and that as regards both these qualities unboiled milk has not been shown to possess any advantage over boiled.

**The Treatment of Intussusception.**—Mr. Edmund Owen[11] considers that there is but one treatment for intussusception, namely, laparotomy and reduction of the incarcerated bowel. The occasional and exceptional success obtained by the injection of air or water is unfortunate, because this treatment leads to the loss of valuable time, and often to injury of the bowel. A discussion on the same subject at the British Medical Association meeting was opened by Mr. Bernard Pitts,[12] who advises inflation only when the case is seen within a few hours of the onset and is not of a very acute character. Hospital cases are much better treated by laparotomy at once. In certain cases there may be an advantage in using inflation for the purpose of reducing the main portion of the intussusception, and enabling the incision to be made directly over the cæcum. When reduction is found to be impossible in chronic cases a resection may be generally done through an incision in the ensheathing bowel. Mr. D'Arcy Power said he had formerly believed in irrigation of the bowel as a suitable method of treatment, but greater experience had taught him that this method was not to be relied on, and he now performed laparotomy at the earliest opportunity. Mr. McAdam Eccles also supported the view that laparotomy was the best treatment, and laid stress on the importance of rapidity in operating, a quarter of an hour being sufficient. After operation he advised small doses of opium to check peristalsis, and spoon feeding in infants, as the act of sucking markedly stimulated the movements of peristalsis.

**An Epidemic of Noma.**—Sixteen cases of this affection are described by Drs. Blumer and Macfarlane[13] as having occurred at the Albany Orphan Asylum after an outbreak of measles which affected 173 of the inmates. Of these 16 cases the mouth alone was affected in four cases, the mouth, ear, and vulva in three cases, the vulva alone was attacked in two of the patients, and in seven along with other parts. The ages of the children affected ranged from three to twelve years. Apart from the measles no cause for the occurrence of noma could be determined, the sanitary surroundings and dietetic arrangements being satisfactory. As a result of bacteriological investigation they conclude that the disease, while originating probably as a simple infection, is always in its later stages a mixed infection, and of the bacteria found they lay stress on a long thread-like organism of the leptothria type. The treatment was stimulating, and when the destruction was not too extensive, thorough cauterisation under chloroform with Pacquelin's cautery seemed to stop the process.

**Lead-poisoning in a Child.**—Dr. G. Variot[14] records a curious case of lead-poisoning from a drinking cup in a child of four and a half years. The patient was brought to the hospital because of increasing loss of power in the limbs, walking having become impossible, and the use of the arms being impaired. There was also some weakness of the flexor muscles of the trunk, and a general diminution of reaction both to faradism and galvanism. Apart from these conditions there was no evidence of nerve lesion, no altered sensibility, no wasting of muscles, and no sphincter trouble. A diagnosis of polyneuritis was made of unexplained origin, there having been no preceding diphtheria or employment of toxic drugs. On further examination the author found a narrow black line on the gums which he was able to pronounce definitely to be from lead poisoning. This led to the employment of iodide of potash and sulphur baths, under which treatment motor power in the limbs was speedily regained. It was noted in this case, as it had been in other cases amongst children, that the lead palsy affected the lower more than the upper extremities, while the converse holds good amongst adult patients. The last point to determine in this case was the source of the poisoning. The home surroundings were thoroughly investigated and symptoms of lead poisoning were looked for in other members of the family, but no clue was discovered until it was learned that the child used a pewter goblet for drinking purposes. The composition of this goblet was analysed with the following result: In 100 grms. there were tin 14·80 grms., antimony 8·73 grms., copper 0·79 grms., and lead 75·68 grms. Further interrogation of the mother elicited the facts that this lead cup had been bought from a hawker, and had been used by the child constantly since he was able to feed himself.

**Posterior Basic Meningitis.**—As regards the ocular symptoms in this affection, Dr. Hugh Thursfield[15] found definite changes in the fundus in thirteen out of seventeen cases. These changes consisted of papillitis with retinal hæmorrhages, or a peculiar grey discolouration of the disc which he has never seen in any other affection, or slight swelling of the disc. Amaurosis was noted in seven cases. Another symptom, which he looks on as characteristic of the disease is retraction of the upper eyelid, which was present in seven cases. The facial appearance is striking as contrasted with that of a child suffering from tuberculous meningitis, for whereas in the latter case there is photophobia and closure of the eyes, in posterior basic meningitis the eyes are widely open with retracted upper lids as if seeking the light.

**Pneumococcic Peritonitis in Children.**—Dr. Charles Michaut[16] has collected and analysed 33 cases of this still little recognised affection. The cause is Frankel's pneumococcus which reaches the peritoneum usually through the blood stream. The primary source in six cases was pneumonia or broncho-pneumonia, and in these the infection was probably carried through the lymphatics. The rarity of this condition as the result of pneumonia is explained as being due to the rapidity with which pneumococci lose their virulence in pulmonary lesions, and also to some extent to the immunisation of the patient. Two varieties of peritonitis are described, encysted and generalised. The former, which occurred 22 times, was usually situated about the umbilicus. The pus is thick and greenish yellow, and the containing membrane is bounded anteriorly by the abdominal wall, and posteriorly by the omentum. As regards the diagnosis, the encysted form may present symptoms very similar to those of tubercular peritonitis, while the generalised form may simulate appendicitis in its early stages.

[7] Lancet, Vol. II., p. 49. [8] Ibid. [9] Ibid., p. 50. [10] Ibid., p. 169. [11] Brit. Med. Jour., Vol. II., 1901, p. 573. [12] Ibid., p. 574. [13] Amer. Jour. Med. Sci., November, 1901. [14] Soc. Med. des Hopit., 1901, No. 30. [15] Lancet, Feb. 16, 1901, p. 460. [16] Gaz. des Hopitaux, 1901, p. 361.

